# Periodontal severity and metabolic syndrome: A cross-sectional study from a tertiary care hospital in India

**DOI:** 10.6026/973206300220477

**Published:** 2026-01-31

**Authors:** Lata Goyal, Amandeep Kaur, Yeshwanth Perambudhuru, Shubham Sareen, Arshad Eranhikkal, Mehak Gupta, Prabhleen Kaur Brar

**Affiliations:** 1 Department of Dentistry, All India Institute of Medical Sciences, Bathinda, Punjab, India; 2 Department of General Medicine, All India Institute of Medical Sciences, Bathinda, Punjab, India; 3 Department of Periodontics, Oral Health Sciences Centre, Postgraduate Institute of Medical Education and Research (PGIMER), Chandigarh, India; 4 Department of Conservative Dentistry, Postgraduate Institute of Medical Education and Research (PGIMER) , Chandigarh, India

**Keywords:** Oral health, hypertension, diabetes, obesity, dyslipidaemia

## Abstract

Periodontitis links to metabolic syndrome (MS) through chronic inflammation affecting teeth-supporting structures. Hence, this cross-sectional study examined 330 metabolic syndrome patients for periodontitis staging, probing depth, attachment loss, teeth count and oral health-related quality of life (OHRQoL). Periodontitis prevalence reached 74.5%, distributed as 44.5% Stage I, 17% Stage II, 7% Stage III and 6% Stage IV; only raised blood sugar correlated significantly (p=0.036). Average OHRQoL score was 20.88±7.42, worsening beyond 35 in Stages III-IV. Findings advance understanding by quantifying staging-specific OHRQoL decline, supporting targeted periodontal screening in metabolic syndrome.

## Background:

Metabolic syndrome (MS) is a cluster of three of five factors, including hypertension, elevated serum Triglycerides and reduced high-density lipoprotein, increased fasting glucose and abdominal obesity, which collectively increase the risk of cardiovascular disease, stroke and diabetes. This combination of conditions can increase the risk of developing thrombotic diseases, impaired resistance to insulin and diabetes mellitus, as well as nervous system complications [1]. Inflammation of the periodontium has a detrimental effect on a patient's systemic health [2]. Papapanou *et al.* re-classified periodontitis in the 2017 World Workshop based on local conditions and systemic risk factors into four stages [3]. Many studies have proven the two-way relationship of periodontitis with diabetes and obesity [4, 5-6]. Microbial component and inflammation are considered the linking factor between periodontitis and systemic conditions. Studies have proven that the pro-inflammatory cytokines from periodontitis can enter circulation and increase generalised inflammation and excess reactive oxygen species, which can contribute to insulin resistance and abnormal endothelial function. Systemic inflammation from MS can also increase oxidative stress in the periodontium, thereby worsening the inflammation [7]. Although few studies noted the link between MS and periodontitis, however, to the best of our knowledge association between the two components in terms of periodontitis staging has not been studied so far. Various stages of periodontal severity differ in terms of treatment, progression and systemic inflammation. Therefore, it is of interest to correlate the severity and complexity of different stages of periodontitis with components of metabolic syndrome, so that more personalised and effective treatment strategies can be implemented and thereby prevent disease progression and potentially reduce the systemic inflammation that impacts overall health.

## Materials and Methods:

## Study design:

The present cross-sectional analysis included 330 adult individuals. Data were obtained from 2022 to 2024 in the Department of Dentistry and General Medicine after taking ethical clearance from the Institutional Ethical Clearance (IEC/AIIMS/BTI/145). Patients having an MS diagnosis in accordance with the Adult Treatment Panel III (ATP III) [8] and who agreed to give consent were included in this study.

[1] Central obesity (waist circumference >102 cm in males and > 88 cm in females).

[2] Hypertension (BP) >130/85 mmHg.

[3] Hyper-triglyceridemia (TG) >150 mg/dL.

[4] Low high-density lipoprotein (HDL) <40mg/dL in males and < 50mg/dL in females.

[5] Fasting blood glucose (FBS) 100 mg/dL.

[6] Patients with diabetes, cardiovascular disease, respiratory disease, pregnant and breastfeeding females were excluded.

Patients with diabetes, cardiovascular disease, respiratory disease, pregnant and breastfeeding females were excluded.

## Sample size:

After screening 1025 patients, the final study sample comprised 330 patients.

Sample size was determined using the formula n = DEFFxNxpx(1-p)/d^2^/(Z^2^_2_-α/2x(N-1)+px(1-p), considering the average prevalence of disease to be 19-29%.

## Data collection:

General information of the participants (age, gender and educational, social status) adapted from BG Prasad were recorded [9]. Two calibrated examiners performed a periodontal examination. Inter examiner reliability was quantified by k, which was 0.9. All the Clinical parameters, including pocket depth and attachment loss, were measured by a Calibrated probe (UNC-15, Hu-Friedy and Chicago, USA). Subjects were grouped as Stage I, II, III and IV based on criteria established by the American Association of Periodontology 2017 guidelines [3]. OHRQoL was measured using the OHIP-14 questionnaire was recorded [10]. The results derived from the present study adhered to reporting standards set by the STROBE guidelines.

## Statistical analysis:

Data was recorded in Microsoft Excel and was evaluated using Statistical Package for the Social Sciences (SPSS) version 23. The data of individuals included in the study were presented with the help of numbers and percentages of categorical variables and median (interquartile range) was used for describing the continuous variables after analysing the data for normality. Assumption of normal distribution was calculated using the Kolmogorov-Smirnov test. Chi-square test/Fisher's exact test was used for assessment of the significance of the association between categorical variables. The difference in distribution of continuous variables across more than two groups was analysed using the Kruskal-Wallis test. Post hoc analysis was also done for multiple pairwise comparisons where necessary; p-value < 0.05 is statistically significant.

## Results:

Three hundred and thirty subjects diagnosed with metabolic syndrome were included. Baseline data were reported in (Table 1). The subjects included had a mean age of 53±11 years (Male, 53±1.9; female, 54±9.63). 52.12% of patients who were minimally educated were affected more with different stages of periodontitis as compared to others (Table 2). 246 individuals (74.5%) were classified as periodontitis patients. The prevalence of all four stages of periodontitis among metabolic syndrome patients and the total number of subjects with different components of MS are summarised in (Table 3). The number of patients having all 5 components of MS was 20.6% while the majority of the patients had 3 or 4 components of MS. Higher prevalence of periodontitis was seen in metabolic syndrome patients having 5 components, 80 % as compared to 75% and 70 % in metabolic syndrome patients having 3 and 4 components, respectively (Table 3). The study's core contribution of comparing individual risk factors of MS with different stages of periodontitis was demonstrated in (Table 4). Which shows a higher percentage of involvement was seen with stage I and II periodontitis among all components. Among all the components, fasting blood sugar revealed a statistically significant relationship with periodontitis (p=0.036), although waist to hip ratio was also associated with periodontitis, but association was insignificant (p=0.155). Hypertension raised serum triglyceride and reduced serum HDL did not show a significant association. A statistically significant association between the periodontitis staging and the Oral Health Impact Profile (OHIP) score was observed. The worst quality of life was seen as the disease advanced (median OHIP increased from 19 in the normal stage to 40 in the Stage 4 periodontitis) (p-value <0.001) (Table 5). Post-hoc analysis showed significant differences in OHIP scores between early stages and advanced stages. Furthermore, weak but positive correlation was seen between higher OHIP scores and DMFT score (ρ=0.165, p=0.003) (Table 5).

## Discussion:

As systemic inflammation is the linking pathway between MS and periodontitis [11]. It is important to consider the severity and staging of periodontitis because a more severe form contributes to systemic inflammation. In the present study prevalence of periodontitis was 74.5 % in the studied group. The majority of them were Stage I (44.5 %), followed by Stage II, III and IV 17 %, 7% and 6 % respectively. Although the periodontitis condition was seen more in patients having 5 components, the association between the three/four components and the severity of periodontitis was insignificant. Among different components of metabolic syndrome, fasting blood sugar (p = 0.036) was significantly associated with periodontitis in the study. Microangiopathy, increased reactive oxygen species, oral microbial flora and impaired defence against antioxidants are some of the factors that dictate the association between diabetes and periodontitis [12, 13]. Consistent with previous research, a statistically significant association (p = 0.036) between blood sugar and periodontitis emphasises the importance of oral health in diabetic individuals [14]. The association of BMI and truncal obesity with periodontitis is attributed to the high levels of inflammatory mediators in the plasma, along with insulin resistance [15]. Statistical association between BMI and stage of periodontitis was established showing that a BMI of 25-29.9 increases the risk of stage III-IV periodontitis by 3.997 times [16, 17]. Venkat & Janakiram showed that BMI and periodontitis are not associated with each other [18]. There was an association between central obesity and periodontitis (p=0.15), but it could not achieve a level of significance; this could be attributed to the comparatively lower percentage of Stage IV periodontitis in the studied group. 93 (88.8%) out of 330 patients were identified with higher serum Triglycerides (>150). Overall, the association between high serum triglycerides and periodontitis was non-significant (p=0.30). In the current study, a surprisingly non-significant association between periodontitis and hypertension was seen (p=0.78) and these findings are consistent with Sakurai *et al.* in 2019 [19]. Although there might be some isolated disparity and controversy in the literature in correlating periodontitis with the individual criteria of the metabolic syndrome alone, the profound influence of the chronic systemic inflammation in all the criteria that collectively define the metabolic syndrome can be traced to the pathogenesis and progression of periodontitis. Additionally, the prevalence of all these criteria together in a patient is much more likely as they have a shared pathogenesis, especially diabetes mellitus, obesity and triglyceride levels. For obese subjects, an increased TG index signals a greater diabetes risk, with factors like sex, age and BMI further influences the risk of diabetes in obese [15]. Understanding these mechanisms could provide insights into how metabolic syndrome may exacerbate periodontitis and vice versa, ultimately improving both disease conditions, which will improve the overall well-being of the individual and eventually enhance the patient's general health and improving their quality of life.

OHRQoL relates patients' oral health to overall well-being. In our study mean score were 20, which suggest that metabolic syndrome patients have an average OHRQoL. But we compare the distribution of OHIP score among different substages of periodontitis; patients having severe periodontitis (III & IV) had a mean of more than 35, which suggests the metabolic syndrome adverse effect on OHRQoL in III & IV periodontitis. Severely affected domains were 1 (functional impairment), 2 (pain) and 5 (mental disability). The worst response was given to question number 2 (Have you noticed decline in food quality and taste recently?), 3 (Do you experience pain in your oral cavity?), 4 (Do you feel stressed while eating?) and 9 (Are you finding it difficult to relax?). There was a positive correlation between the number of DMFT, but this was weak, suggesting that the greater the DMFT score, adverse the effect on the OHRQoL. It is well known that the progression of periodontitis is inevitable in the absence of prompt intervention. And in their lifetime, there is always a risk of remission of the disease even in treated patients, which mainly depends on various modifiable and unmodifiable factors. This study describes the complex interactions between metabolic syndrome and periodontitis, both biologically and in terms of how it affects patients' quality of life with different degrees of inflammation. The rationale behind the idea of using periodontitis staging as the screening tool in metabolic syndrome is to prevent the risk of progression of disease in patients with inflammatory risk factors who neglect their oral hygiene. So, comprehensive longitudinal studies utilising a large sample size will be crucial to understand these complex relationships. The current study is cross-sectional, which observed an association at one point in time. A longitudinal study with multiple follow-ups would be required to determine whether MS patients develop severe periodontitis or vice versa. Other factors like stress, genetics, diet, lifestyle and occupation, which have a relation with systemic conditions, were beyond the scope.

## Conclusion:

Diabetes, followed by abdominal obesity, showed the strongest and significant associations with periodontitis. Screening for metabolic syndrome in periodontal patients and vice versa could improve early diagnosis and prompt management. Periodontitis staging may serve as a valuable adjunctive marker in identifying individuals at risk for systemic disease.

## Figures and Tables

**Table 1:** Depicts demographic data of the patients

		**Count**	**N %**
Sex	Female	144	43.60%
	Male	186	56.40%
Education	Primary	172	52.12%
	Secondary	134	40.60%
	Higher	24	7.27%
Socioeconomic status	Lower (L) class	117	35.50%
	Middle (M) class	200	60.60%
	Upper (U) class	13	3.90%
BMI	<30	233	70.60%
	>30	97	29.40%

**Table 2:** Depicts levels of social class with different stages of periodontitis

**Parameter**	**Education**			**Socioeconomic status**		
	**1o**	**2o**	**3o**	**L**	**M**	**U**
Normal	11.21%	11.51%	2.12%	8.48%	16.96%	0.60%
Stage I PD	23.02%	19.09%	1.81%	20%	22.72%	2.12%
Stage II PD	9.39%	6.36%	1.21%	3.93%	12.42%	0.30%
Stage III PD	4.84%	2.12%	1.21%	1.81%	4.54%	0.60%
Stage IV PD	3.63%	1.51%	0.90%	1.21%	3.93%	0.30%

**Table 3:** Distribution of stages of periodontitis among metabolic syndrome patients

		**N (%)**	**Severity of Metabolic Syndrome**			**p-value**
			**3 components**	**4 components**	**5 components**	
Periodontitis	Non P	84 (25.5%)	32 (24.2)	38(29.2)	14(20.6)	0.683
	Stage1	147 (44.5%)	64(48.5)	51(39.2)	32(47.1)	
	Stage2	56 (17%)	20(15.2)	22(16.9)	14(20.6)	
	Stage3	23 (7%)	7(5.3)	12(9.2)	4(5.9)	
	Stage4	20 (6.1%)	9(6.8)	7(5.4)	4(5.9)	

**Table 4:** Depicts the portion of subjects and the association between components of metabolic syndrome and periodontitis with metabolic syndrome parameters

**Parameter**	**Threshold Limit**	n	**%**	**Non Period**	**Stage I**	**Stage II**	**Stage III**	**P value**	**n**
Abdominal obesity	Normal	12	3.63%	1(8.3)	5(41.7)	2(16.7)	2(16.7)	2(16.7)	0.155
	Obese	318	96.36%	83(26.1)	142(44.7)	54(17)	21(6.6)	18(5.7)	
High blood pressure	>130/80	284	86.10%	9(19.6)	22(47.8)	7(15.2)	4(8.7)	4(8.7)	0.781
	<130/80	46	13.90%	75(26.4)	125(44)	49(17.3)	19(6.7)	16(5.6)	
Hyper-triglyceridemia	>150mgldl	293	88.80%	74(25.3)	130(44.4)	53(18.1)	18(6.1)	18(6.1)	0.334
	<150mg/dl	37	11.20%	10(27)	17(45.9)	3(8.1)	5(13.5)	2(5.4)	
Low high-density lipoprotein	<40	153	46.40%	39(25.5)	65(42.5)	27(17.6)	10(6.5)	12(7.8)	0.75
	>40	177	53.60%	45(25.4)	82(46.3)	29(16.4)	13(7.3)	8(4.5)	
High fasting glucose	≥100	208	63.00%	47(22.6)	94(45.2)	35(16.8)	21(10.1)	11(5.3)	0.036
	<99	122	37.00%	37(30.3)	53(43.4)	21(17.2)	2(1.6)	9(7.4)	
Number of components of Metabolic Syndrome	3	132	40.00%						
	4	130	39.40%						
	5	68	20.60%						

**Table 5:** Distribution of OHIP among different stages of Periodontitis/Relationship between OHIP and Periodontitis and correlation with DMFT

	**Normal**	**Stage 1**	**Stage 2**	**Stage 3**	**Stage 4**	**p-value**
Overall OHIP Score	19(14-23)	20(15-25)	22(14-25)	37(30-40)	40(33-45)	<0.001
			**Median number of DFMT (IQR)**			
OHIP score≤35			2(1-5)			
OHIP score>35			5(4-7)			
Spearman Correlation Between OHIP score and DFMT: ꝭ=0.165, p-value=0.003						
